# Citrus-Based Bio-Insect Repellents—A Review on Historical and Emerging Trends in Utilizing Phytochemicals of Citrus Plants

**DOI:** 10.1155/jt/6179226

**Published:** 2024-11-28

**Authors:** S. S. S. T. Fernando, R. G. P. T. Jayasooriya, Kalpa W. Samarakoon, N. D. Asha D. Wijegunawardana, Sampath B. Alahakoon

**Affiliations:** ^1^Institute for Combinatorial Advanced Research and Education (KDU-CARE), General Sir John Kotelawala Defence University, Kandawala Road, Dehiwala-Mount Lavinia, Sri Lanka; ^2^Department of Bioprocess Technology, Faculty of Technology, Rajarata University of Sri Lanka, Mihintale, Sri Lanka; ^3^Department of Chemistry, Faculty of Science, University of Peradeniya, Peradeniya, Sri Lanka

**Keywords:** citrus, essential oils, insect repellency, micro/nano-encapsulation, phytochemicals

## Abstract

Research on citrus plants is the result of increasing interest in the discovery of plant species with potential insect-repellent properties. Insect-repelling ability can be achieved by the numerous ubiquitous citrus species. This is mainly due to the presence of phytochemicals such as limonene, citronellol, citral, and *α*-pinene. These phytochemicals' composition varies depending on the geographical location of the plant. The extraction method dictates the configuration of attainable phytochemicals while the dosage affects the repellency potential. Therefore, developing insect repellent involved a number of observations related to the identification of both citrus plant phytochemical composition present in the different parts of the plant and the repellency potential of these phytochemicals in advance. Conversely, the development of repellent methods that go beyond conventional methods has been made possible by scientific developments including modern strategies such as encapsulation, the preparation of emulsion, and the incorporation of repellents into textiles. Therefore, this review article intends to probe into the aforementioned information and provide a sound insight into citrus-based repellent development in the future.

## 1. Introduction

Insects, the most diverse and widespread group of animals on Earth, inhabit nearly all terrestrial ecosystems. In these ecosystems, they play various roles as predators, parasites, and most importantly, as pathogens and vectors of disease. Insects not only have important roles in sustaining life but can also have a direct impact on public health by transmitting diseases to humans and animals. Stinging insects in particular are considered pests and can act as disease vectors [1]. The impact of insects on humans is multifaceted, as they can cause infections and vector-borne diseases and damage fruit and vegetable crops and livestock. According to the World Health Organization (WHO), more than 17% of all infectious diseases and more than 700,000 deaths per year are due to vector-borne diseases [2]. These diseases are transmitted by insect vectors, which can be divided into two categories. Mechanical vectors, such as cockroaches, various flies, and other non-hematophagous insects, transmit the pathogen mechanically through physical contact. Biological vectors, on the other hand, are hematophagous, such as mosquitoes, ticks, and fleas, and transmit the pathogen to the host through a blood meal [1]. Therefore, interrupting transmission plays a key role in controlling the disease spread. This could be achieved through vector control or repellency. However, vector control can interfere with the natural balance of the ecosystem negatively affecting its biodiversity, whereas insect repellency is a sound alternative for vector-borne disease control.

Insect repellents function by interacting with insect's odorant receptors (ORs) and gustatory receptors (GRs), which alter insect physiology and behavior [3]. The odor molecules are bound to odor-binding proteins (OBPs) in the insect antenna, which causes their displacement, according to the hypothesized mechanism [4]. Furthermore, volatile compounds comprise odorants with the capacity to either suppress or activate olfactory receptor neurons (ORNs). By preventing insects from biting their hosts, these chemicals act as insect repellents. As tastants, nonvolatile substances, on the other hand, have the power to either excite or repress gustatory receptor neurons (GRNs) [5].

In nature, plants possess various natural compounds called phytochemicals that serve as effective defenses against phytophagous insects. These compounds exhibit remarkable abilities to repel, poison, degrade food, or regulate the growth of these insect species [6]. These active compounds are classified according to factors such as chemical structure, composition, solubility, and synthesis pathways and form several families, including phenols, terpenes and steroids, alkaloids, and flavonoids [7]. Phytochemicals, as a complex cocktail of active ingredients, exhibit dual action against insects, by targeting both their behavior and physiological processes [8]. This exceptional property gives them high efficiency as natural insect repellents and minimizes the likelihood of insects developing specific resistance. Because of this advantage and the realization that synthetic repellents are less environmentally friendly and pose greater toxicity risks to humans, the use of natural plant phytochemicals in the manufacture of insect repellents has increased.

Many plants have been used by people as insect repellents since ancient times, dating back to ancient Greece [9]. Numerous plants have been explored over the years as potential sources of pesticides and repellents. However, the U.S. Environmental Protection Agency (U.S. EPA) has officially approved only a small number of plants. Citronella, lemon, and eucalyptus oils are among these certified plants; they are preferred by manufacturers because of their comparatively low toxicity and proven efficacy. Therefore, they are commonly used in natural repellents [10].

## 2. Insect Repellency Mechanism

Repellency by definition is a broader spectrum including various forms of behavior orientations between the repellent and the insect. Such different types of repellencies include (i) true repellency/spatial repellency, which results in the insect moving directly away from the source without intimate contact, (ii) landing inhibition/excito-repellency, caused by movement of the insect after being in direct contact with the repellent compound, (iii) antifeedant/suppressant repellency, which causes interference with feeding activity after being in contact with the repellent compound, (iv) odor-masking/attractant inhibition repellency, which works by hindering the attraction of the insect toward a specific host by negatively influencing the locating ability of the host, thus practically making the host scent undetectable by odorant signal, and (v) visual masking, which negatively affects the ability of the insect to identify the host through visual signals [11]. Out of the types mentioned above, true repellency is considered expellant, where insects move away from the odor source [10]. For instance, (E)-*β*-farnesene, an aphid alarm-pheromone found in citrus plants, prompts dispersal in aphids, demonstrating true repellency [12]. Similarly, sesquiterpene found in leaf and peel extracts of several citrus plant species [13, 14] also indicates the true repellency.

**Figure 1:**
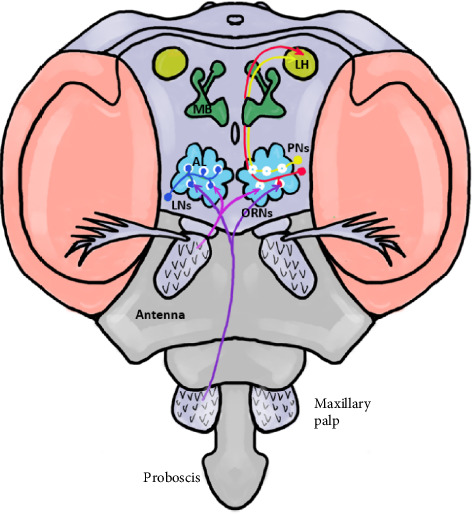
Olfactory pathway of insects (*Drosophila*). The sensilla in the antenna and maxillary palp of insects direct the olfactory receptor neurons (ORNs) into the antenna lobe (AL); this signal is perceived by multi-glomerular local interneurons (LNs). These signals are then taken into the mushroom body (MB) and lateral horn (LH) by the projection neurons (PNs).

**Figure 2:**
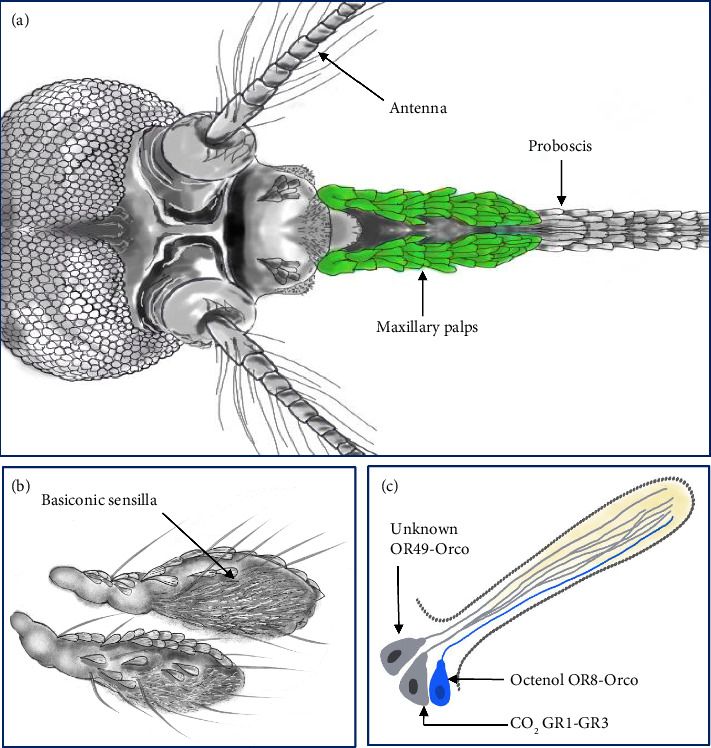
Olfactory system of the female *Aedes aegypti* mosquito. (a) Three olfactory appendages are present on the dorsal view of the *A. aegypti* head: the proboscis, maxillary palps (colored green in this image), and antennae. (b) Basiconic sensilla are the sole chemosensory organs found on the fourth segment of the maxillary palps. (c) Diagram illustrating the conceptual neural structure of a basiconic sensillum, which contains three chemosensory.

Insects respond to stimuli mainly through olfactory and gustatory pathways. The olfactory system (Figures 1 and 2) detects volatile chemical signals [15]. Odorant molecules bind to proteins and interact with olfactory receptors, activating neurons and transferring sensory information to the brain [16, 17].

By understanding the basic mechanism in which olfactory senses are perceived by insects, we can get a clear idea of how the olfactory pathway–related repellent mechanisms take place. First is the hypothetical true repellent (expellant) mechanism, where sensory signals from repellants activate specific glomeruli and projection neurons, causing insects to move away [11]. The next type is hypothetical odorant masking repellency, in which the compounds are not repellents like in expellant repellency, but rather have the power to reduce the host's attraction to the insect. In hypothetical odorant masking repellency mechanisms, high concentrations of odorant molecules activate more olfactory receptors, altering neuron signals and resulting in varied responses [11]. In the putative attractant inhibition mechanism, the chemicals actively disrupt the ORN response by inhibiting odorants, binding proteins, and olfactory receptors. This hinders signal transduction, resulting in the insects' capacity to detect the unique host.

Aside from the sense of smell, insects, like vertebrates, respond favorably to sweet taste stimuli and negatively to bitter stimuli. This is achieved via a gustatory response mechanism. Insect gustatory organs are scattered throughout the body, rather than exclusively the head. Chemicals enter GRs, leading to neuron depolarization and signal transmission to the brain [11, 18, 19]. There are various hypotheses on insect repellent systems that use gustatory circuits. In the hypothetical antifeeding mechanism, substances stimulate specific GRs, causing deterrent cells to trigger antifeeding behavior [20]. The hypothetical irritant mechanism is different from the antifeeding mechanism such that the insect actively moves away from the host after coming in contact with the repellent source. Here, the repellent compounds activate GRs on tarses (legs), leading to signal transmission and away movement [11]. Understanding these mechanisms helps in comprehending how insects perceive and react to repellents.

## 3. Citrus Plant Active Compounds

The genus *Citrus* L. belongs to the subtribe Citrineae, which is part of the tribe Citreae within the subfamily Aurantioideae of the family Rutaceae [21]. The genus *Citrus* includes about 60 species, of which about 10 are used in agriculture in which Southern and Southeastern Asia is the main region of the origin of citrus and most *Citrus* species [22]. These plants have traditionally been used as natural deterrents, and in-depth research has shown that the phytochemicals of citrus fruits of various species have the potential for insect repellency. This has led to the commercial availability of these compounds to consumers. Different *Citrus* species have different essential oil (EO) compositions, but they all share some constituents in varying amounts. Moreover, even within a single *Citrus* species, the different vegetative parts have different phytochemical profiles. Therefore, it is crucial to develop a thorough understanding of the phytochemical composition of different *Citrus* species, taking into account their growth in wild, semiwild, and cultivated states over different seasons around the world. Factors such as seasonal variations, geographic location, harvesting period, soil composition, and other environmental conditions significantly impact the oil quality, extraction efficiency, and compositional consistency across different citrus species [23].

The EOs of citrus fruits have an interesting composition as far as secondary plant compounds are concerned. They consist of over 200 components, including both volatile and nonvolatile fractions. According to the authors, these oils can be considered as examples of medium complexity. The volatile fraction, which accounts for 85%–99% of the total oil, consists of a variety of substances, including aliphatic aldehydes, alcohols, and esters, as well as monoterpene and sesquiterpene hydrocarbons and their oxygenated derivatives. The nonvolatile residue, which constitutes 1%–15% of the oil, consists of hydrocarbons, sterols, fatty acids, waxes, carotenoids, coumarins, psoralens, and flavonoids [24]. Citrus monoterpenes include hydrocarbons like *d*-limonene, *γ*-terpinene, *p*-cymene, *β*-phellandrene, and *β*-pinene, as well as oxygenated compounds like geranial, nonanal, and (Z)-neral [25]. Citrus EO's volatile fractions contain terpene alcohol (e.g., linalool, terpin 4-ol, citronellol, and *α*-terpineol), sesquiterpenes (e.g., *γ*-elemene, humulene, and germacrene D), aldehydes (e.g., undecanal), and esters (e.g., sabinene hydrate, linalyl acetate, and neryl acetate) [26].

Due to the presence of many bioactive compounds, citrus secondary metabolites have numerous useful properties. Citrus flavonoids play an important function in controlling oxidative stress, aiding inherent antioxidant properties. The presence of varied flavonoids (e.g., naringin, naringenin, hesperidin, and rutin) [27] and especially ascorbic acid accounts for the antioxidant properties of citrus [28, 29]. Inflammation and discomfort are typical indications of various disorders. Anti-inflammatory drugs help to modulate inflammatory pathways by reducing the production of proinflammatory cytokines and enzymes. The presence of flavonoids, terpenoids, steroids, glycosides, alkaloids, carotenoids, and phenolic compounds is responsible for the anti-inflammatory activity [30–32] and analgesic activity [33, 34] of citrus. The presence of flavonoids and terpenoids aids in regulating anxiety by altering the gamma-aminobutyric acid (GABA) receptors. Citrus plants contain compounds such as rutin, quercetin, kaempferol, and myricetin which are responsible for anxiolytic activity [30, 35]. Apart from these, citrus secondary metabolites are also used as neuroprotective [36], cardiovascular [37], and anticancer [38] treatments. The presence of flavonoids and terpenoid derivatives such as linalyl acetate, linalool, limonene, and *γ*-terpinene is highly useful in promoting the antimicrobial activity of citrus plants [39–41]. Aside from the innate bioactive capabilities of citrus, the current review will go into detail on the insect-repellent potential of different citrus plant species and how they affect different types of insects.

Numerous studies have been conducted on the phytochemicals of citrus fruits, and each of these studies uses a slightly different technique to extract the active ingredients. To increase their efficacy, it is crucial to separate these bioactive compounds from the crude extracts. Fractionation is often performed based on the acidity, polarity, or molecular size of the extracted chemicals [42]. The most widely used extraction technique is hydrodistillation using the Clevenger apparatus, which largely extracts the water-soluble EO component of citrus secondary metabolites. Depending on the solvent used, other extraction techniques such as maceration and Soxhlet extraction are also used to obtain certain polar or nonpolar active compounds [43] (Table 1). Therefore, the choice of extraction method may also affect the relative abundance and identity of the extracted active compounds.

Both gas chromatography (GC) and liquid chromatography (LC) are commonly used in the analysis of volatile and nonvolatile fractions of phytochemicals. Although GC is often used to analyze volatile components, LC is the preferred technique for efficient isolation of compounds with larger molecules. In accurate identification of compounds, GC and LC are often coupled with mass spectrometry (MS). Taken together, the mass spectra and the chromatographic peaks allowed unambiguous identification of each component. Examination of the available GC-MS profiles of numerous citrus species, from a variety of sources and collected at different times, reveals a recurrent trend. The chemical profiles of most citrus species consistently contain two to three prominent components. Depending on variables such as location and other environmental factors, the identities of these dominant chemicals may change within a species [23]. Depending on the plant part studied, the composition and major chemical compounds extracted from citrus plants vary widely. This diversity makes it difficult to accurately calculate the percentage abundance of chemicals. Despite this complexity, some notable compounds stand out clearly from the others.

**Figure 3:**
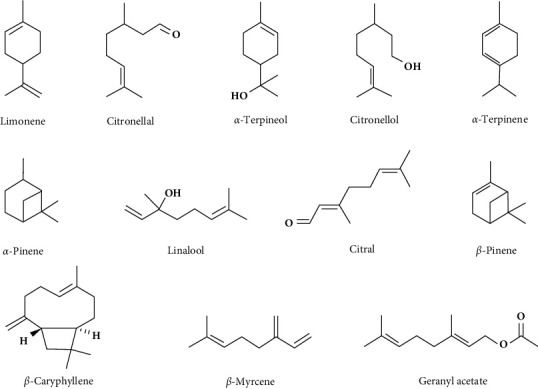
Chemical structures of commonly available phytochemicals in citrus plants. The most common chemical compounds in citrus plants are phenolics and terpenoids out of which hydrocarbons and derivate mono- and sesquiterpenes constitute majority of volatiles.

Over 140 unique chemicals entities have been found in various citrus plants through numerous GC-MS profiling studies (Table 2). Based on their structural makeup, these compounds can be broadly classified into monoterpenes, sesquiterpenes, aromatic compounds, linear compounds (such as fatty acids, aldehydes, and alkanes), and others. In particular, monoterpenes and sesquiterpenes can be divided into oxygenated monoterpenes and sesquiterpenes and monoterpene/sesquiterpene hydrocarbons. Among these substances, some phytochemicals are particularly abundant in various citrus species. For example, limonene is an important constituent found in most citrus species. Pinene, *β*-caryophyllene, *β*-myrcene, terpinene, and citral are some other notable substances regularly found in GC-MS data of citrus species (Figure 3). In the next section, a summary of several reported constituent profiles from different citrus species is provided.

### 3.1. *Citrus sinensis*

The chemical composition of orange peel EOs consists mainly of monoterpene hydrocarbons (58%), oxygenated monoterpenes (31.0%), and sesquiterpene hydrocarbons (9.70%) of which 41 compounds account for 99.84% of the total composition [59]. Main constituents in the *Citrus sinensis* peel includes limonene, *β*-myrcene, and *α*-pinene [59, 60] *β*-pinene and linalool [60]. Additionally, it contains trace amounts of phytochemicals, each comprising less than 0.25% of the total composition, such as sabinene, 1,8-cineole, *α*-phenandrene, *α*-thujene, *γ*-terpinene, *cis*-ocimene, borneol, and *α*-terpineol [59]. In contrast to other peel extracts, significant differences in phytochemical amounts were found when the chemical composition of the leaf extracts was analyzed. The amount of limonene (5.02%) was much lower than that detected in the peels which is between 92% and 94%. Other substances such as *β*-pinene, *α*-fenchocamphorone, *α*-terpineol, *α*-terpinene, and E-*β-*ocimene were detected in higher concentrations than limonene [56].

### 3.2. *Citrus grandis* (Synonym *Citrus maxima*)

A recent study in India used GC-MS profiling method to analyze extracts obtained from *Citrus maxima* leaves and peels, which revealed the presence of more than 45 active compounds [44]. The study revealed that quantities of these compounds varied depending on the specific part of the plant examined. The leaf extract of *C. grandis* contains a total of 42 components with *β*-caryophyllene, (−)-spathulenol, and citronellol being the three most abundant compounds. Several other components, such as *τ-*cadinol, 1-Ethenyl-1-methyl-2-(1-methylethenyl)-4-(1-methylethylidene) cyclohexane, *α*-caryophyllene, 1-bromo-4-bromomethyl decane, and 2-n-hexylcyclopentanone, are also present in relatively high quantities [44]. The fruit peel extract contained a total of 34 compounds, with limonene comprising the majority of the total composition. The remaining three principal constituents in the peel extract are *β*-copaene, *β*-myrcene, and *β*-pinene [44, 55]. Remaining chemicals were found only in small amounts less than 1%. None of the identified chemicals were present in both the leaf and peel extracts even though they were of same origin.

### 3.3. *Citrus hystrix*

*Citrus hystrix* consists of a few major monoterpene compounds; one study successfully identified multiple organic volatile components by extracting the volatile fraction from the medium polar fiber of *Citrus hystrix* fruit peel using headspace-solid phase microextraction (HS-SPME). The main volatile components detected were 3-carene, citronellal, d-limonene, *α*-pinene, *α*-cadinene, copaene, linalool, caryophyllene, and *γ*-Cadinene [61]. Contradictorily, the major compounds found in the leaf extracts are different to those of peels as examined in a different study resulting in 29 distinct components, with a significant presence of oxygenated monoterpenes. These oxygenated substances constituted approximately 86.15% of the total oil. Among them, *β*-citronellal was the predominant monoterpenoid of the leaf oil. Citronellol, linalool, and *β*-citronellol were also detected in the leaf oil. The remaining components comprised less than 2% of the oil composition [46].

### 3.4. *Citrus medica*

The volatile fraction of *Citrus medica* fruit peel comprises a total of around 40 active compounds. Among these, d-limonene, citral, and *β*-myrcene are the primary components [57, 58]. Other notable compounds include linalool, caryophyllene, *α*-pinene, *β*-cubebene, *γ*-cadinene [57], *β*-myrcene, neryl acetate, and neryl alcohol [58]. A study conducted in Bangladesh examined the EOs derived from the leaves and peels of *Citrus medica*. The leaf EO contained erucylamide, limonene, citral, 3,7-dimethyl-, acetate, (Z)-, 6-octenal, 3,7-dimethyl-, 1,2-cyclohexanediol, 1-methyl-4-(1-methylethenyl)-, and methoprene as the most abundant constituents. The analysis reveals that both the leaf and peel EOs of *Citrus medica* exhibit a complex mixture of compounds of which many were present only in trace amounts. It is important to highlight that the chemical composition of the EOs derived from the leaves and peels of *C. medica* displays significant variations [58].

### 3.5. *Citrus aurantifolia*

*Citrus aurantifolia* leaf oil contains a total of roughly 31 distinct compounds, whereas its peel oil contains approximately 26 different compounds. The primary constituents of *Citrus aurantifolia* leaf EO were identified as citral and limonene. On the other hand, limonene and palatinol-1C were found to be the main components in the oil extracted from the peel. Both oils also contained limonene and farnesol [63].

### 3.6. *Citrus aurantium*

*Citrus aurantium* peel, leaf, and flower samples have been studied for their phytochemical properties. It was found that the EOs extracted from flowers and leaves contained a significant proportion of oxygenated monoterpene compounds (59.02%–69.21%). The primary components within this group were linalool and linalyl acetate. Furthermore, hydrocarbon monoterpene compounds were present at notable levels, with *α*-thujene and *β*-pinene being the most abundant. The EO extracted from peels predominantly consisted of the hydrocarbon monoterpene limonene [13, 47] and in lesser quantities *β*-ocimene, *β*-pinene, and *β*-myrcene [47]. Oxygenated monoterpene components accounted for only 11.68% of the total oil [13]. Additionally two sesquiterpene hydrocarbons, namely, *β*-caryophyllene and *β*-farnesene, are also present in notable amounts. Nonterpenic chemicals were also found, with alcohols comprising 0.52% of the total composition, aldehydes 1.26%, and esters 0.8% [47].

### 3.7. *Citrus limon*

Lemon leaf EO consists of around 28 volatile components, constituting 99.5% of the entire oil composition. *Citrus limon* leaf oil exhibited a high concentration of alcohol molecules (61.55%), followed by esters (24.92%) and monoterpenes (12.10%). Linalool consists of the major fraction with geraniol, *α-*terpineol, and linalyl acetate as the primary constituents of lemon leaf EO. Additionally, geranyl acetate, neryl acetate, *β*-pinene, and *cis*-ocimene were recognized as significant components [64]. *C. limon* peels constitutes 43 chemical substances. Limonene and neral were the major compounds discovered, while *tran*s-verbenol exhibited an intermediate concentration. Several minor constituents have also been identified, including decanal, ethyl cinnamate, ethyl *p*-methoxycinnamate, *cis*-*α*-bergamotene, geraniol, *trans*-carveol, nonanal, linalool, *α*-terpineol, *p*-mentha-2,8-dien-1-ol, estragole, and *α*-fenchene [48].

### 3.8. *Citrus limetta*

A major fraction of *C. limetta* peel is monoterpene hydrocarbons of which more than 95% is of limonene [14, 65], a principal constituent found in nearly all citrus oils. Among the other constituents detected, camphene was the second most abundant compound following limonene. Additionally, *ρ*-cymene, geraniol, *α*-terpinene, *α*-terpineol, neral, and *β*-bisabolene were present. Furthermore, minor quantities (below 0.1%) of citronellal, geraniol, *α*-pinene, *β*-pinene, *α*-thujene, and *α*-humulene were identified [65].

## 4. Insect Repellent Activity

As explained in the previous section, citrus plants possess a broad spectrum of active phytochemical constituents with each characterized by distinct concentrations and percentage compositions. The intricate makeup of these bioactive compounds allows citrus plants to effectively serve as potent insect repellents against a diverse array of insect species, including mosquitoes, ticks, mites, beetles, and flies. The complex blend of compounds found in citrus varieties presents a challenge for different insect species to develop specific resistance mechanisms against these active constituents. This phenomenon is supported by numerous scientific investigations conducted on citrus plants, which have demonstrated that the repellent properties against insects vary based on the specific combination of compounds present [66].

The measurement of insect repellence activity can be done in many different ways. The most common and recommended mode is through the measurement of percentage repellency which uses the below given formula:

(1) $$\begin{array}{c} \text{Percentage Repellency }\left(\text{PR}\right)=\left[\frac{\left(\text{Nc}-\text{Nt}\right)}{\left(\text{Nc}+\text{Nt}\right)}\right]*100, \end{array}$$

where Nc is the number of insects present in the negative control and Nt is the number of insects present in the treatment.

This method is being used in both mosquito and insect repellency measurement. As per the WHO guidelines for efficacy testing of spatial repellents, an appropriate statistical analysis should include data of the number of replicates of the control and treatment groups, the length of the test, the duration of protective efficacy, and the mean percentage of landing inhibition or feeding inhibition with a 95% confidence interval [67].

(2) $$\begin{array}{c} \text{Percentage landing inhibition}=\left[\frac{\left(\text{Cl}-\text{Tl}\right)}{\text{Cl}}\right]*100, \end{array}$$

where Cl is the number of mosquitoes landing in the control space and Tl is the number of mosquitoes landing in the treatment space.

(3) $$\begin{array}{c} \text{Percentage feeding inhibition}=\left[\frac{\left(\text{Cf}-\text{Tf}\right)}{\text{Cf}}\right]*100, \end{array}$$

where Cf is the number of blood fed mosquitoes in the control space and Tf is the number of blood fed mosquitoes in the treatment space.

Depending on the percentage repellency, the following sextet classification scale is used to classify averages into the given categories: (0 to V) Class as shown in Table 3 [68].

When evaluating the insect-repelling properties of various citrus plants based on the predominant secondary metabolites they contain, the effectiveness in repelling different types of insects varies. The identical secondary metabolite may exhibit varying degrees of repellent action against distinct insect species. *Citrus aurantium*, with its primary active compound being d-limonene, has demonstrated effective repellent properties against the two-spotted spider mite (*Tetranychus urticae*) [69]. Natural oils and a synthetic blend of monoterpenes and sesquiterpenes closely resembling one of the natural oils have exhibited similar repellent effects for up to 3 h. Notably, during the evaluation period, natural *C. aurantium* peel oil displayed a higher repellent efficacy (90.56 ± 5.80%) when compared to synthetic oils (64.61 ± 1.56%). When assessing the impact of individual compounds on *T. urticae*, the synergistic combination of various prominent secondary metabolites, such as *d*-limonene, terpinolene, and citronellal, outperformed isolated single compounds like *α*-terpineol (90.52 ± 3.88%), *d*-limonene (71.33 ± 3.08%), citronellal (78.99 ± 2.34%), and eugenol (62.00 ± 3.83%) [69]. Due to variations in the percentage composition of secondary metabolites depending on the plant part's location, leaf extracts of *Citrus aurantium* have also shown to be effective against other insects, including the sawtoothed grain beetle (*Oryzaephilus surinamensis* F.), cigarette beetle (*Lasioderma serricorne* L.), and rice weevil (*Sitophilus oryzae* L.), with repellency percentages of 96.66%, 73.33%, and 78.33% for each, respectively. Based on repellency percentages, these results fall within sextet classification classes V, IV, and IV, respectively [70]. The study found that limonene in *Citrus aurantium* affected *Tribolium castaneum* in a way that did not depend on the concentration of the substance, after a two-hour exposure period. Interestingly, this effect was found to be concentration-dependent after one hour, except at concentrations of 50 and 100 μL/L air. Moreover, the repellency class remained constant in this time frame [47].

*Citrus sinensis* var. pera oils have shown satisfactory repellency against insects such as the two-spotted spider mite *(Tetranychus urticae)* [69], *Aedes aegypti* [71, 72], *Anopheles stephensi* [72], *Culex quinquefasciatus* [71, 72], and *Zabrotes subfasciatus* [73]. The most common secondary metabolite identified during the analysis was *d*-limonene. The extracts have shown to be good mosquito repellents with repellency classes ranging from IV to V (70%–90%) [71, 72], whereas the repellency against other insects lies slightly in the less moderate range between classes of less than III [69, 73]. The repellency percentage notably increases with higher dosage concentrations; however, the optimal dosage for effective repellent production may not necessarily be the highest dosage but rather the one that achieves higher repellency with a lower dosage [74].

EO extracts from *Citrus aurantifolia* against *Ae. aegypti* [71, 75] and *Cx. quinquefasciatus* [71] have revealed a high repellency of Class V with less concentrations. Limonene was identified as the most prominent compound, followed by *β*-pinene [75]. In the case of *C. aurantifolia*, all EOs showed stronger activity as feeding inhibitors compared to their repellent activity against mosquito species. This effect may result from the activation of bitter GRNs upon the detection of active compounds by the labellum, or from the inhibition of sweet GRNs following the same stimulus [5]. Apart from the gustatory responses, *C. aurantifolia* shows significant olfactory responses in cockroach species such as *Blattella germanica, Periplaneta americana,* and *Periplaneta* fuliginosa. This dual form of repellency by *C. aurantifolia* could be due to synergistic effect of *γ*-terpinene, *β*-pinene, *α*-pinene, and limonene at approximately equal parts [76].

Exposure of *T. castaneum* and *C. maculatus* to extracts of *Citrus maxima* has shown a mean repellent activity of more than 50% (Class III) and 78.7% (Class IV), respectively, within 2–24 h after exposure [54]. Depending on the insect's stage in life cycle, a compound's repellent capability varies at a given concentration. This difference can be seen when *Citrus maxima* EOs showed only minimal repellent effects on juvenile *C. maculatus*, but significant repellent activity (varying from Class II to Class IV) against adult *C. maculatus* during 2–24 h of exposure [54]. Furthermore *C. maxima* has also been tested for repellency against mosquito species such as *Ae. aegypti* [71, 77] and *Cx. quinquefasciatus* [71]. In both cases, *Citrus maxima* exhibited remarkable repellency, exceeding 94% and categorizing as Class V repellency. Among various citrus plant species, *C. maxima* predominately contains limonene as a major secondary metabolite. Consequently, this limonene content is presumed to be a key factor contributing to the strong repellent activity of *C. maxima* against mosquito species.

*Citrus hystrix* was shown to have the potential to mitigate the negative effects of *C. maculatus*, particularly by significantly reducing seed damage and weevil perforation index (31.01) while increasing the percentage of protective effect (68.98%) without affecting seed germination [61]. Unlike most of the other citrus species, *Citrus hystrix* has citronellal as the most abundant active secondary metabolite which has satisfactory repellency against common mosquito species such as *An. minimus* [78], *Ae. aegypti* [71, 78], and *Cx. quinquefasciatus* [71] from both leaf and peel extracts.  Table 4 illustrates the relationship between the citrus species and the repellency on specific insect species along with the dosage utilized.

## 5. Emerging Trends in Utilizing Citrus Plant Phytochemicals for Insect Repellency

Numerous conventional applications utilize the phytochemicals found in various plant species, including citrus, for their insect repellent properties. Insect repellent creams, sprays, incense sticks, coils, and vaporizers have been produced and used for years. The prevailing trend now is to use natural products derived from plants because of their low toxicity and high biosafety in repelling insects. Consequently, there is a growing interest in researching and improving the efficacy of these phytochemicals through innovative approaches and improvements to existing techniques.

### 5.1. Encapsulation

**Figure 4:**
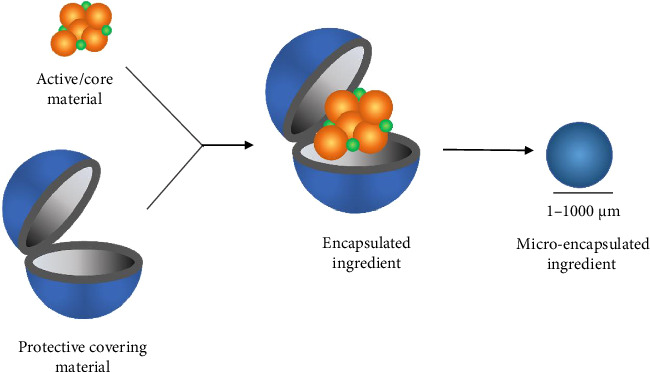
Microencapsulation procedure. The figure depicts the microencapsulation process, in which bioactive substances are enclosed in tiny capsules of protective covering material for gradual release. As seen in the image, this process results in the creation of microcapsules that are uniform in size and morphology. The resulting microcapsules successfully shield the bioactive compounds from deterioration and permit continuous release, making them appropriate for use in a variety of pharmaceutical, food, and cosmetic applications.

Although natural phytochemicals generally have a higher percentage of repellents compared to synthetic repellents such as N, N-diethyl-*meta*-toluamide (DEET), their efficacy decreases significantly after about 3 h of exposure to the external environment. In contrast, DEET can maintain its repellent activity for about 6–8 h [84]. The main limitation of natural plant-based repellents, such as citrus, is that they cannot retain the active ingredients on the treated surfaces. Although the addition of vanillin as a binder can somewhat extend the retention time, this is not a long-term solution [85]. To solve this problem, encapsulation techniques can be used to extend the retention time of the active ingredients. Encapsulation involves the production of uniform, micro- or nanoscale spherical capsules with a core containing the encapsulated substance and a wall or shell surrounding it (also referred to as a coating or membrane) (Figure 4). Various encapsulation techniques are commonly used, including coacervation, spray drying, centrifugal extrusion, fluidized bed coating, freeze drying, emulsion, liposome entrapment, spray cooling, solvent evaporation, and in situ polymerization [86]. The composition of the polymeric wall, such as its density, permeability, and biological properties, influences the release of constituents from the capsule, which in turn affects the rate at which the compounds are released. In addition, external environmental factors also play an important role in the release of core constituents from the capsules, including mechanical, chemical, thermal, and diffusion stimuli [87].

A scientific investigation was undertaken to assess the efficacy of *Citrus grandis* oil as a repellent against *Ae. aegypti* mosquitoes and to evaluate its impact on mosquito longevity. The findings revealed that the microencapsulated formulation consistently exhibited superior repellent attributes, even after a 1-year storage period. As a result, it enabled the sustenance of a high level of protection (> 80%) for an additional 4 h of exposure. Conversely, the nonencapsulated form offered effective protection for only 1 h following application. Preserving the stability of the formulation through microencapsulation proved to be pivotal in ensuring enduring protection beyond a 12-month storage duration [88].

### 5.2. Insect Repellent Fabrics

In recent years, there have been numerous studies investigating the use of insect repellents in fabrics and clothing designed primarily for outdoor activities. To achieve an optimal finish, insect repellent textiles are commonly created by carefully applying repellent chemicals to the fabric in its final form [89]. Ongoing research conducted by international scientists is driving the development of textiles with mosquito-repellent properties [90]. It is important to introduce binder compositions that can adhere to fabrics and support the repellent agents to ensure effective adhesion of the agents to the fabrics. Insect-repellent fabrics can also be proactively produced by incorporating repellent chemicals into the fiber or yarn before material preparation, particularly using synthetic fibers where insect repellents are sprayed on the fiber during the spinning process [89]. When considering the primary material for fabric manufacturing, the textile industry heavily relies on cotton, a natural plant fiber [91]. Additionally, repellent properties can be imparted to other fabric materials such as polyester [92] and nylon [93]. Due to regular washing, textiles are subjected to extensive wear and tear. Consequently, repellent materials used in these textiles tend to diminish after multiple wash cycles. To improve their durability on the textile surface, a varnishing process is employed. However, repellent chemicals may leak out during subsequent washing cycles due to poor affinity between repellents and textiles or insufficient attachment to the textile fibers [94]. The techniques for incorporating active citrus compounds into the core fabric material mentioned above vary depending on the specific protocol used.

#### 5.2.1. Microemulsification

Microemulsions are homogenous mixtures of two mutually insoluble liquids, such as oil and water, that are translucent. Surfactants or a combination of surfactant and co-surfactant molecules support these combinations, successfully stabilizing the system [95]. EOs can be effectively microemulsified to enhance the shelf life of the final product while maintaining the functional and physicochemical properties of the oil. Cotton samples impregnated with the litsea-lemon EO blend (in a ratio of 1:2) stabilized in chitosan-sodium alginate polyelectrolyte assemble displayed a repellency of 52.3 ± 4.2% (mean ± SD) while cotton samples treated with a microemulsion had a mean repellency of 72.9 ± 13.9% (mean ± standard deviation (SD)) against *Ae. aegypti* mosquitoes 24 h after treatment according to a recent study. This EO mixture contained *Citrus limon* with major compounds of limonene and citral according to GC-MS profiling. These results depict that EOs that have been micro-emulsified can adhere to textile fibers, potentially broadening their range of uses beyond simple one-time treatments [96].

#### 5.2.2. Micro/Nano-Encapsulation Method

In a study conducted by Zayed et al., extracts from the peel of *Citrus sinensis* were nano-encapsulated using silver and zinc oxide nanoparticles to develop a multifunctional cotton fabric where the extracts from *Citrus sinensis* peels were prepared using both water and ethanol [97]. It was followed by the synthesis of nanoparticles and an embedding to the cotton fabric using chitosan, a naturally occurring polymer obtained from chitin by chemical deacetylation [98]. The findings of the study showed that the treated cotton fabrics exhibited toxicity to mosquitoes (*Ae. aegypti*). Rates of mosquito repellency, knockdown, and mortality also increased with increasing exposure time. Interestingly, the treated fabrics exhibited a stronger toxic effect on insects than untreated fabrics, even though the washing process slightly reduced the residual toxicity of the treated fabrics [97].

#### 5.2.3. Direct Application of Natural Repellents by Pad-Dry-Cure Method

Herbal extracts from the peel of *Citrus sinensis* (sweet lime) were applied to cotton fabric using the pad-dry-cure technique [91]. The efficacy of the resulting substance in repelling mosquitoes was then investigated. The findings indicate a positive correlation between the duration of padding and the mosquito repellent properties of the fabric. The best results were obtained at a 60% extract concentration for a duration of 120 min, resulting in an 80% repellency rate. Moreover, the repellent finishes exhibited remarkable durability even after undergoing six washing cycles in a domestic setting, thus highlighting their robustness.

## 6. Conclusion and Future Outlook

Escalating global temperatures have triggered a continuous proliferation of insects across diverse ecosystems paving roads to a rise in demand for insect repellents. Natural plant-based repellents due to their superior levels of customer satisfaction and safety compared to synthetic alternatives have witnessed a growing presence in the market. Thus, it is imperative to explore potential plant species with insect repellent properties. Several citrus species distributed worldwide exhibit remarkable repellent properties primarily due to phytochemicals such as limonene, citronellol, citral, and pinene. However, the repellent activity of citrus species may depend on factors such as the location of the plant, extraction methods, and extract dosage employed. Scientific advances have facilitated the development of repellent solutions that go beyond traditional sprays, fumigants, coils, and creams. Modern innovations include strategies such as encapsulation, the production of emulsions, and infusion of repellents into textiles that provide long-term efficacy. Despite the challenges, these modern innovative strategies together with the use of phytochemicals from plants such as citrus are essential for the development of efficient insecticidal and repellent treatments for effective indoor and outdoor insect pest control. This review focuses primarily on the use of citrus plants and highlights promising and available findings regarding their efficacy in repelling various insect pests.

## Figures and Tables

**Table 1:** Extraction methods used in obtaining citrus active compounds.

Method of extraction	Common compounds extracted	References
Hydrodistillation (Clevenger's apparatus)	Sesquiterpene hydrocarbons, oxygenated monoterpene, monoterpenes hydrocarbon, and nonaromatic and aromatic hydrocarbons	[13, 44–48]
Soxhlet extraction	Monoterpene, monoterpenoid ester	[45, 49–52]
Cold-pressing method	Monoterpene hydrocarbons, aliphatic and sesquiterpene hydrocarbons, carbonyl compounds, and alcohols.	[53]
Pressurized liquid extraction	Monoterpene hydrocarbon, oxygenated monoterpene, sesquiterpene hydrocarbon, polyunsaturated fatty acid, triterpenes	[49]
Supercritical CO_2_ extraction	Mono and poly-unsaturated fatty acids	[50]
Supercritical fluid extraction	Monoterpene hydrocarbons, oxygenated monoterpenes, sesquiterpene hydrocarbons	[51]

**Table 2:** Percentage composition of active compounds in different citrus plants.

	Active compound	*Citrus grandis*		*Citrus medica*		*Citrus sinensis*		*Citrus hystrix*		*Citrus aurantifolia*		*Citrus aurantium*			*Citrus limon*		*Citrus limetta*
		Peel (%)	Leaves (%)	Peel (%)	Leaves (%)	Peel (%)	Leaves (%)	Peel (%)	Leaves (%)	Peels (%)	Leaves (%)	Leaves (%)	Flowers (%)	Peels (%)	Peels (%)	Leaves (%)	Peels (%)
		[44, 54, 55]	[44, 56]	[57, 58]	[56, 58]	[53, 59, 60]	[56]	[61]	[46, 62]	[45]	[63]	[13]	[13]	[13, 47]	[48, 60]	[64]	[14, 65]
Monoterpene	*d*-Limonene	89.04–93.20	3.00	68.31	18.36	92.14–94.30	5.02	11.54	—	44.59	11.59	6.52	5.03	73.60–87.52	55.40–59.3	1.13	78.30–95.98
	*α*-Pinene	0.41–4.32	0.91	0.41–1.73	0.43	0.50–0.70	0.11	9.24	—	—	—	0.68	0.77	0.10–0.56	0.17–2.90	0.20	0.04–1.58
	3-Carene	1.49	—	—	—	—	—	18.31	—	—	—	—	—	—	0.34	0.11	—
	Cyclohexene, 4-methylene-1-(1-methylethyl)	0.40	2.66	—	—	—	—	—	—	—	11.33	—	—	—	—	—	—
	*trans*-Linalool oxide (furanoid)	1.17	—	—	—	—	—	—	—	—	—	—	—	—	—	—	—
	*cis*-Linaloloxide	0.62	—	—	—	—	—	—	0.24	—	—	—	—	—	—	—	—
	1,6-Octadien-3-ol, 3,7-dimethyl	0.76	—	—	4.39	—	—	—	—	—	—	—	—	—	—	—	—
	2,6-Octadienal, 3,7-dimethyl-, (z)	0.31	—	—	—	—	—	—	—	—	—	—	—	—	—	—	—
	2-Carene	0.26	—	—	—	—	—	—	—	—	—	—	—	—	—	—	—
	Sabinene	—	—	1.41	—	0.11–0.20	—	—	0.20–0.44	—	—	≤ 0.1	≤ 0.1	0.18	0.60	0.41	0.08
	*β*-Pinene	2.25	—	—	—	0.02–1.30	20.69	—	—	—	—	9.68	9.21	0.40–0.90	13.4	4.51	5.60
	*cis*-Ocimene	—	—	—	—	0.12	—	—	—	—	—	—	—	—	—	2.93	—
	*β*–Myrcene	2.06–2.90	—	2.70–7.59	—	1.57–2.70	—	—	0.08	—	—	≤ 0.1	≤ 0.1	0.90–1.60	—	—	0.08
	Allo ocimene	—	—	—	—	0.07	—	—	—	—	—	—	—	—	—	—	—
	*α*-Thujene	—	—	—	—	0.19	—	—	—	—	—	10.65	6.15	≤ 0.1	—	—	0.07
	Fenchone	—	—	—	—	0.13	—	—	—	—	—	—	—	—	—	—	—
	*γ-*Terpinene	—	—	0.30	—	0.23	—	0.94	—	0.12	—	≤ 0.1	≤ 0.1	≤ 0.1	8.60	0.05	—
	Isoartemisia	—	—	—	—	0.05	—	—	—	—	—	—	—	—	—	—	—
	1,8-Cineole	—	—	—	—	0.33	—	—	—	—	—	—	—	—	—	—	—
	*α-*Terpinene	—	—	—	0.25	1.50–1.70	7.45	—	—	—	—	0.80	0.72	≤ 0.1	—	0.03	0.33
	*α*-Fenchene	—	—	—	—	—	—	—	—	—	—	1.21	≤ 0.1	≤ 0.1	0.46	—	—
	Camphene	—	—	—	—	—	—	—	—	—	—	1.92	1.92	≤ 0.1	—	—	0.06–1.78
	*δ*-3-Carene	—	—	—	—	—	—	—	—	—	—	≤ 0.1	≤ 0.1	0.33	—	—	—
	*α*-Phellandrene	—	—	—	—	—	—	—	—	—	—	0.24	≤ 0.1	≤ 0.1	—	—	—
	(z)-*β*-Ocimene	0.33	—	—	33.03	—	—	0.46	—	—	—	≤ 0.1	0.36	≤ 0.1	—	—	—
	(E)-*β*-Ocimene	—	—	—	—	—	5.02	—	—	—	—	≤ 0.1	0.12	1.12	—	—	—
	*o*-Cymene	—	—	—	—	—	—	—	—	—	—	≤ 0.1	≤ 0.1	≤ 0.1	—	—	—
	*p*-Cymene	—	—	—	—	—	—	—	—	—	—	≤ 0.1	≤ 0.1	≤ 0.1	—	—	—
	*cis*-Linalool oxide	—	—	—	—	—	—	—	—	—	—	0.59	0.28	≤ 0.1	—	0.07	—
	Terpinolene	—	—	—	—	—	—	—	—	0.13	—	≤ 0.1	≤ 0.1	0.15–0.21	0.60	—	—
	Camphor	—	0.65	—	—	—	—	—	—	—	—	—	—	—	0.34	—	—
	Pinocarvone	—	—	—	1.76	—	—	—	—	—	—	—	—	—	—	—	—
	*α*-Fenchocamphorone	—	—	—	2.76	—	18.62	—	—	—	—	—	—	—	—	—	—
	Myrcene	—	—	—	—	—	—	—	—	—	—	—	—	—	3.50	—	—
	1,8 Cineole	—	—	—	—	—	—	—	—	—	—	—	—	—	—	—	0.76
	Cyclohexanone, 2-methyl-5-(1-methylethenyl)	—	—	—	2.24	—	—	—	—	—	—	—	—	—	—	—	—
	Isolimonene	—	—	39.37	—	—	—	—	—	—	—	—	—	—	—	—	—
	*cis*-2,6-Dimethyl-2,6-octadiene	—	—	—	—	—	—	2.22	0.33	—	—	—	—	—	—	—	—

Monoterpene alcohol	*β*-Terpineol	—	—	—	—	—	—	2.69	—	0.41	—	—	—	—	—	—	—
	Nerol	—	7.00	—	—	0.08	—	—	—	—	—	≤ 0.1	0.34	0.83	0.4	—	—
	Linalool	0.49	—	4.78	—	0.05–0.60	—	4.02	3.90	5.06	—	37.33	41.23	3.30–4.80	1.16	30.62	5.15
	*trans*-Linalool	—	—	—	—	—	—	—	—	—	—	3.88	0.17	≤ 0.1	—	—	—
	Borneol	—	—	—	—	0.17	—	0.27	—	—	—	—	—	—	—	—	0.01
	Citronellol	—	28.26	—	—	—	3.52	1.32	1.76–11.48	9.72	—	—	—	—	—	—	—
	*α*-Terpineol	0.57	—	0.52	—	—	12.1	—	0.11	2.26	—	≤ 0.1	1.26	0.12	—	14.52	0.31–0.51
	*cis*-Sabinene hydrate	—	—	—	—	—	—	—	—	—	—	≤ 0.1	≤ 0.1	≤ 0.1	—	0.07	—
	*trans-p*-Menth-2-en-1-ol	—	—	—	—	—	—	—	—	—	—	3.10	≤ 0.1	≤ 0.1	—	—	—
	Terpinen-4-ol	—	0.19	—	—	—	—	—	0.34	—	—	≤ 0.1	0.40	0.07	0.17	0.43	0.03
	*cis-p*-Menth-2-en-1-ol	—	—	—	—	—	—	—	—	—	—	≤ 0.1	≤ 0.1	≤ 0.1	—	—	—
	Geraniol	—	—	—	13.36	—	—	0.47	0.42	11.96	10.59	≤ 0.1	2.46	≤ 0.1	0.60–1.48	15.91	0.36
	Genariol	—	—	—	—	—	1.37	—	—	—	—	—	—	—	—	—	—
	*β*-Citronellol	—	—	—	—	—	—	—	6.59	—	—	—	—	—	—	—	—
	*β*-Linalool	—	—	—	—	—	—	—	1.65	—	3.50	—	—	—	—	—	—
	*trans*-Verbenol	—	—	—	—	—	—	—	—	—	—	—	—	—	6.43	—	—

Monoterpene ester	Geranyl acetate	0.64	—	—	—	—	—	—	1.80	1.80	—	1.70	2.49	≤ 0.1	0.40	6.75	—
	Borneol acetate	—	—	—	—	0.12	—	—	—	—	—	—	—	—	—	—	—
	Linalyl acetate	—	—	—	—	0.13	—	—	—	—	—	7.87	13.75	1.60	—	13.76	—
	Citronellyl acetate	—	—	—	—	—	—	—	—	—	—	≤ 0.1	≤ 0.1	≤ 0.1	—	—	—
	*α*-Terpinyl acetate	—	—	—	—	—	—	—	—	—	—	≤ 0.1	2.57	1.38	—	—	0.06
	Neryl acetate	—	—	2.51	—	0.06	—	—	—	2.42	—	≤ 0.1	1.01	0.51	0.70	4.24	—
	Neryl formate	—	—	—	20.52	—	—	—	—	—	—	—	—	—	—	—	—
	Norborneol acetate	—	1.55	—	—	—	—	—	—	—	—	—	—	—	—	—	—
	Acetate de bornyle	—	—	—	4.49	—	—	—	—	—	—	—	—	—	—	—	—
	Acetate de sabinyle	—	—	—	5.23	—	—	—	—	—	—	—	—	—	—	—	—
	Lsobornyl propanoate	—	—	—	5.01	—	—	—	—	—	—	—	—	—	—	—	—
	Geraniol acetate	—	—	—	—	—	—	—	—	—	5.82	—	—	—	—	—	—

Monoterpene aldehyde	Neral	—	—	0.81	—	0.09	—	—	—	—	—	3.40	4.80	0.75–3.26	1.30–10.39	—	0.28–0.30
	Geranial	0.20	10.04	—	1.23	0.07	3.02	0.14	—	—	—	0.19	0.28	0.10–0.42	1.60	—	0.03–0.21
	Citral	0.37	—	23.12	12.95	—	—	—	—	—	13.46	—	—	—	—	—	—
	*β*-Citronellal	—	—	—	—	—	—	—	66.85–85.43	—	2.83	—	—	—	—	—	—
	Citronellal	—	—	0.64	0.22	—	—	12.68	—	4.07	—	0.21	≤ 0.1	≤ 0.1	0.12	—	0.07–0.10

Sesquiterpene	*α*-Cubebene	3.18	—	1.47	2.04	—	—	—	—	—	—	—	—	—	—	—	—
	*β*-Cubebene	—	—	1.69	—	—	—	—	—	—	—	—	—	—	—	—	—
	Caryophyllene	0.41	2.48	1.78	—	—	—	3.99	—	—	—	—	—	—	—	—	—
	*α*-Guaiene	0.90	—	—	—	—	—	0.14	—	—	—	—	—	—	—	—	—
	*β*-Caryophyllene	—	16.89	—	—	0.12	—	—	—	—	—	1.00	0.45	0.55	0.60	—	—
	*β*-Elemene	—	0.74–3.62	—	—	0.03	—	—	—	—	—	0.21	≤ 0.1	≤ 0.1	—	0.70	—
	*δ*-Elemene	—	—	1.50	—	—	—	—	—	—	—	1.61	≤ 0.1	≤ 0.1	—	—	—
	*α*-Copaene	—	—	—	—	—	—	4.30	—	—	—	≤ 0.1	≤ 0.1	0.60	—	—	—
	*trans-β*-Bergamotene	—	—	—	—	—	—	—	—	—	—	0.16	≤ 0.1	≤ 0.1	—	—	—
	*γ*-Elemene	—	—	0.44	—	—	—	—	—	—	—	2.62	0.29	1.21	—	—	—
	(E)-*β*-Farnesene	—	—	—	—	—	—	—	—	—	—	0.10	≤ 0.1	≤ 0.1	—	—	0.03
	*α*-Humulene	—	—	—	—	—	—	—	—	—	—	≤ 0.1	≤ 0.1	0.37	—	—	—
	*β*-Bisabolene	—	—	—	—	—	—	—	—	—	—	≤ 0.1	≤ 0.1	0.17	—	—	0.10
	Bicyclogermacrene	—	—	—	—	—	—	—	—	—	—	≤ 0.1	≤ 0.1	≤ 0.1	—	—	0.03
	(E,E)-*α*-Farnesene	—	—	—	—	—	—	—	—	—	—	≤ 0.1	≤ 0.1	2.58	—	—	—
	*δ*-Cadinene	—	—	—	—	—	—	—	—	—	—	0.16	≤ 0.1	0.87	—	—	—
	*α*-Cadinene	—	—	—	—	—	—	4.30	—	—	—	—	—	—	—	—	—
	*β*-Copaene	—	—	—	—	—	1.82	—	—	—	—	—	—	—	—	—	—
	Acorenone B	—	8.46	—	—	—	0.95	—	—	—	—	—	—	—	—	—	—
	Germacrene A	—	—	1.78	—	—	—	—	—	—	—	—	—	—	—	—	—
	*δ*-Cadinene	—	—	1.60	—	—	—	—	—	—	—	—	—	—	—	—	—
	*γ*-Cadinene	—	—	—	—	—	—	3.18	—	—	—	—	—	—	—	—	—
	*γ*-Muurolene	—	—	—	—	—	—	3.07	—	—	—	—	—	—	—	—	—
	*cis-α-*Bergamotene	—	—	—	—	—	—	—	—	—	—	—	—	—	1.60	—	—

Sesquiterpene alcohol	(E)-Nerolidol	—	—	—	—	—	—	—	—	—	—	≤ 0.1	1.12	0.40	—	—	—
	(E,Z)-Farnesol	—	—	—	—	—	—	—	—	—	—	≤ 0.1	≤ 0.1	0.58	—	—	0.03
	Fokienol	—	1.35	—	—	—	0.25	—	—	—	—	—	—	—	—	—	—
	*β*-Bisabolenol	—	1.53	—	—	—	—	—	—	—	—	—	—	—	—	—	0.10
	Spathulenol	—	9.23	—	—	—	—	—	—	—	—	—	—	—	—	—	—
	*τ*-Cadinol	—	3.16	—	—	—	—	—	—	—	—	—	—	—	—	—	—
	*α*-Cadinol	—	2.51	—	—	—	—	—	—	—	—	—	—	—	—	—	—

Sesquiterpene aldehyde	*β*-Bisabolenal	—	3.02	—	—	—	0.25	—	—	—	—	—	—	—	—	—	—

Sesquiterpene ester	(E,Z)-Farnesyl acetate	—	—	—	—	—	—	—	—	—	—	0.13	0.29	0.63	—	—	—

Sterol	*β*-Sitosterol	17.99	—	—	—	—	—	—	—	—	—	—	—	—	—	—	—
	*α*-Sitosterol	12.19	—	—	—	—	—	—	—	—	—	—	—	—	—	—	—
	Stigmasterol	5.22	—	—	—	—	—	—	—	—	—	—	—	—	—	—	—
	Desmosterol	3.79	—	—	—	—	—	—	—	—	—	—	—	—	—	—	—
	Campesterol	4.31	—	—	—	—	—	—	—	—	—	—	—	—	—	—	—
	4,4-Dimethyl cholesta-22,24-dien-5-ol	0.82	—	—	—	—	—	—	—	—	—	—	—	—	—	—	—
	(3á,22E) 3-Methoxy-stigmasta-5,22-diene,	1.95	—	—	—	—	—	—	—	—	—	—	—	—	—	—	—
	24-Propylidene-, (3á) cholest-5-en-3-ol	1.90	—	—	—	—	—	—	—	—	—	—	—	—	—	—	—
	9,19-Cyclolanost-24-en-3-ol	2.41	—	—	—	—	—	—	—	—	—	—	—	—	—	—	—

Steroid	Allopregnane-3á,7à, 11à-triol-20-one	0.39	—	—	—	—	—	—	—	—	—	—	—	—	—	—	—

Alcohol	Octen-3-ol	—	7.71	—	—	—	—	—	—	—	—	—	—	—	—	—	—
	3-Undecanol	—	—	—	—	—	—	—	1.04	—	—	—	—	—	—	—	—
	Dictamnol	—	2.91	—	—	—	—	—	—	—	—	—	—	—	—	—	—

Aliphatic alcohols	Decanol	—	—	—	—	—	—	—	—	—	6.19	—	—	—	—	—	—

Ester	Ethyl cinnamate	—	—	—	—	0.14	—	—	—	—	—	—	—	—	—	—	—
	Hexyl acetate	—	—	—	—	—	—	—	—	—	—	≤ 0.1	≤ 0.1	≤ 0.1	—	—	—
	Methyl anthranilate	—	—	—	—	—	—	—	—	—	—	0.12	0.29	0.63	—	—	—
	Furfuryl d'acetate	—	—	—	3.49	—	—	—	—	—	—	—	—	—	—	—	—

Volatile organic compound (VOC)	Jasmone	—	—	—	—	—	—	—	—	—	—	0.16	≤ 0.1	≤ 0.1	—	—	—
	Z-Trimenal	—	—	—	2.96	—	—	—	—	—	—	—	—	—	—	—	—
	3-Hexen-1-ol	—	—	—	—	—	—	—	0.03	—	—	—	—	—	—	—	—

Aldehyde	Nonanal	—	—	—	—	—	—	—	—	—	—	0.25	≤ 0.1	≤ 0.1	1.19	—	0.01
	Nonenal	—	2.12	—	—	—	2.77	—	—	—	—	—	—	—	—	—	—
	Decanal	0.20	—	1.23	—	0.22	—	0.40	—	—	—	—	—	—	3.25	—	—

Ketone	6-Methyl-5-hepten-2-one	—	—	—	—	—	—	—	—	—	—	≤ 0.1	≤ 0.1	≤ 0.1	—	—	—

Polycyclic aromatic hydrocarbons (PAHs)	*α-*Phenandrene	—	—	—	—	0.14	—	—	—	—	—	—	—	—	—	—	—

Aromatic hydrocarbon	*α-p-*Dimethylstyrene	—	—	—	—	—	—	—	—	—	—	≤ 0.1	≤ 0.1	≤ 0.1	—	—	—
	Pentyl-benzene	—	1.35	—	—	—	2.46	—	—	—	—	—	—	—	—	—	—

Volatile aromatic compounds	2-Phenylethyl	—	—	—	—	—	—	—	—	—	—	≤ 0.1	≤ 0.1	≤ 0.1	—	—	—

Benzopyrone	Coumarin	—	—	—	2.34	—	—	—	—	—	—	—	—	—	—	—	—

Phenylpropene	Eugenol	—	—	—	—	—	1.62	—	—	—	—	—	—	—	—	—	—

Saturated hydrocarbon	*cis*-Decahydro-naphthalene	—	16.09	—	—	—	—	—	—	—	—	—	—	—	—	—	—

Alkaloid	Indole	—	—	—	—	—	—	—	—	—	—	0.5	0.1	≤ 0.1	—	—	—

Fatty acid derivatives	2,4-Dodecadienoic acid, 11-methoxy-3,7,11-trimethyl-, methyl ester, (E,E)	—	—	—	1.22	—	—	—	—	—	—	—	—	—	—	—	—

Fatty acid amide	Erucylamide	—	—	—	28.43	—	—	—	—	—	—	—	—	—	—	—	—

**Table 3:** Sextet classification scale is used to classify the percentage repellency.

Class	% repellency	Repellency
0	0.01–0.1	Low repellency
I	0.1–20.0	
II	20.1–40.0	

III	40.1–60.0	Moderate repellency
IV	60.1–80.0	

V	80.1–100	High repellency

**Table 4:** Percentage repellent activity of citrus plants on different insect species.

Plant	Part used	Insect repelled	Dosage	% repellency	Reference
*Citrus sinensis Osbeck* var. pera	Fruit	*Tetranychus urticae*	1% MeOH solution of EO	43.35%	[69]
*Citrus aurantium* L	Fruit	*Tetranychus urticae*	1% MeOH solution of EO	90.56%	

*Citrus nobilis*	Peels	*Callosobruchus maculatus*	7 g	86.67%	[79]
*Citrus medica*	Peels	*Callosobruchus maculatus*	7 g	65.00%	

*Citrus medica*	Leaves	Culicidae		> 75%	[80]

*Citrus aurantium* L	Leaves	*Lasioderma serricorne* (F.)	0.1–0.83 μL/cm^2^	73.33%	[70]
*Citrus aurantium* L	Leaves	*Oryzaephilus surinamensis* (L.)	0.1–0.83 μL/cm^2^	96.66%	
*Citrus aurantium* L	Leaves	*Sitophilus oryzae* (L.)	0.1–0.83 μL/cm^2^	78.33%	

*Citrus aurantifolia*	Fruit	*Aedes aegypti*	50%	96.60%	[75]

*Citrus limonum*	Peel	*Anopheles stephensi*	1%	92.63%	[81]

*Citrus maxima*	Peel	*Tribolium castaneum*	0.5 mg/cm^3^	53.30%	[54]
			1.5 mg/cm^3^	58.70%	
			2.5 mg/cm^3^	65.30%	
			3.5 mg/cm^3^	73.30%	
			5.0 mg/cm^3^	78.70%	
*Citrus maxima*	Peel	*Callosobruchus maculatus*	0.5 mg/cm^3^	23.90%	
			1.5 mg/cm^3^	38.70%	
			2.5 mg/cm^3^	49.30%	
			3.5 mg/cm^3^	65.30%	
			5.0 mg/cm^3^	69.30%	

*Citrus aurantifolia*	Fruit	*Aedes aegypti*	0.33 μL/cm^2^	98.53%	[71]
*Citrus aurantium* L.	Fruit			98.27%	
*Citrus hystrix* DC.	Fruit			98.53%	
*Citrus maxima* (Burm.f.) Merr.	Fruit			97.73%	
*Citrus medica* L. var sarcodactylis Swingle	Fruit			98.54%	
*Citrus reticulata* Blanco	Fruit			98.67%	
*Citrus sinensis* Osbeck	Fruit			98.78%	
*Citrofortunella microcarpa* (Bunge) Wijnands	Fruit			98.40%	
*Citrus aurantifolia*	Fruit	*Culex quinquefasciatus*	0.33 μL/cm^2^	98.27%	
*Citrus aurantium* L.	Fruit			98.57%	
*Citrus hystrix* DC.	Fruit			98.33%	
*Citrus maxima* (Burm.f.) Merr.	Fruit			98.67%	
*Citrus medica* L. var sarcodactylis Swingle	Fruit			98.67%	
*Citrus reticulata* Blanco	Fruit			98.57%	
*Citrus sinensis* Osbeck	Fruit			98.67%	
*Citrofortunella microcarpa* (Bunge) Wijnands	Fruit			68.57%	

*Citrus grandis*	Peel	*Aedes aegypti*	5%	52.00%	[77]
			10%	62.50%	
			20%	94.70%	

*Citrus sinensis*	Peel	*Anopheles stephensi*	50 ppm	74.60%	[72]
			150 ppm	79.20% (after 2 h)	
			250 ppm	87.60%	
			350 ppm	91.41%	
			450 ppm	100%	
		*Aedes aegypti*	50 ppm	72.80%	
			150 ppm	74.20%	
			250 ppm	83.40%	
			350 ppm	87.60%	
			450 ppm	98.20%	
		*Culex quinquefasciatus*	50 ppm	64.60%	
			150 ppm	72.20%	
			250 ppm	82.20%	
			350 ppm	85.40%	
			450 ppm	91.00%	

*Citrus hystrix*	Peel	*Callosobruchus maculatus*	7 g	19.60%	[61]

*Citrus hystrix*	Leaf	*Anopheles minimus*	2% v/v	87.20%	[78]
		*Aedes aegypti* (L.)	2.5% (v/v)	63.30%	
	Peel	*Anopheles minimus*	5% (v/v)	96.28%	
		*Aedes aegypti* (L.)	2.5% (v/v)	46.50%	

*Citrus reticulata*	Peel	*Callosobruchus maculatus*	7 μL	40.00%	[82]
*Citrus limon*	Peel	*Callosobruchus maculatus*	7 μL	36.66%	
*Citrus aurantium*	Peel	*Callosobruchus maculatus*	7 μL	36.66%	

*Citrus aurantium*	Leaves, flowers, peels	*Tribolium castaneum*	100 μL/L	64.00%	[13]

*Citrus medica*	Peel	*Callosobruchus maculatus*	7 g	65.00%	[57]

*Citrus sinensis*	Peel	*Zabrotes subfasciatus*	0.3 g	53.75%	[73]

*Citrus paradisi*		*Blattella germanica*	10 μL	96.70%	[76]
*Citrus limonum*		*Periplaneta americana*	10 μL	92.60%	
*Citrus aurantifolia*		*Periplaneta fuliginosa*	10 μL	86.70%	

*Citrus medica*	Leaves	*Tribolium castaneum*	78.63 nL/cm^3^	92.00% (after 2 h)	[83]
				94.00% (after 4 h)	
	Fruits	*Tribolium castaneum*	78.63 nL/cm^3^	82.00% (after 2 h)	
				90.00% (after 4 h)	

## Data Availability

The primary research data supporting this review article are from previously reported studies and datasets, which have been cited promptly within the article.
